# Screening and patient-tailored care for emotional and cognitive problems compared to care as usual in patients discharged home after ischemic stroke (ECO-stroke): a protocol for a multicenter, patient-blinded, cluster randomized controlled trial

**DOI:** 10.1186/s12913-020-05902-2

**Published:** 2020-11-17

**Authors:** J. P. L. Slenders, R. M. Van den Berg-Vos, C. M. van Heugten, J. M. A. Visser-Meily, S. M. A. A. Evers, R. J. de Haan, J. M. de Man-van Ginkel, V. I. H. Kwa

**Affiliations:** 1 Department of Neurology, Amsterdam UMC, Amsterdam, the Netherlands; 2 Department of Neurology, OLVG, Amsterdam, the Netherlands; 3 Department of Neuropsychology & Psychopharmacology, Faculty of Psychology and Neuroscience (FPN), Maastricht University, Maastricht, the Netherlands; 4 School for Mental Health & Neuroscience, Department of Psychiatry & Neuropsychology, Faculty of Health, Medicine and Life Sciences (FHML), Maastricht University Medical Center, Maastricht, the Netherlands; 5 Department of Rehabilitation, Physical Therapy Science & Sports, UMC Utrecht Brain Center, University Medical Center Utrecht, Utrecht, the Netherlands; 6 Center of Excellence for Rehabilitation Medicine, UMC Utrecht Brain Center, University Medical Center Utrecht, and De Hoogstraat Rehabilitation, Utrecht, the Netherlands; 7 Department of Health Services Research, Maastricht University, Care and Public Health Research Institute (CAPHRI) of the Faculty of Health, Medicine and Life Sciences, Maastricht, the Netherlands; 8 Trimbos Institute, Netherlands Institute of Mental Health and Addiction, Centre of Economic Evaluation & Machine Learning, Utrecht, The Netherlands; 9 Clinical Research Unit, Amsterdam University Medical Center, Amsterdam, The Netherlands; 10 Department of Nursing Science, Julius Center for Health Science and Primary Care & UMC Utrecht Brain Center, University Medical Center Utrecht, University Utrecht, Utrecht, The Netherlands

**Keywords:** Ischemic stroke, Screening, Cognitive and emotional problems, Randomized controlled trial, Cost-effectiveness, Process evaluation

## Abstract

**Background:**

Ischemic stroke patients with a good outcome in terms of motor functioning and communication are likely to be discharged home without further rehabilitation. A significant number of these patients experience cognitive and emotional problems resulting in lower quality of life and decreased participation in society. This paper presents the protocol of a study examining the clinical effectiveness, cost-effectiveness and implementation of an intervention focused on screening and patient-tailored care for cognitive and emotional problems as compared to usual care in patients discharged home after ischemic stroke.

**Methods / design:**

A multicenter, patient-blinded, cluster randomized controlled trial will be performed. Centers will be randomized (1:1) to the intervention group or the usual care group. Patients (> 18 years old) with a neurological confirmed diagnosis of ischemic stroke who can be discharged home without follow-up treatment at an outpatient rehabilitation clinic will be included. In the intervention group, patients will receive a short, individualized, semi-structured consultation by specialized nurses in addition to usual care. This consultation includes 1) screening for cognitive and emotional problems, 2) screening for restrictions in participation, 3) promotion of self-management strategies and 4) a decision tool for referral to rehabilitation services. The intervention will be performed approximately 6 weeks after the stroke at the neurology outpatient clinics and will take approximately 60 min. The control group will receive care as usual. Both groups will be followed-up at 6 weeks, 3 months and 12 months after stroke. The primary outcome will be the level of participation measured with the Restriction subscale of the Utrecht Scale for Evaluation of Rehabilitation on the level of Participation (USER-Participation-R) at 12 months. A cost-effectiveness analysis and process evaluation will be performed alongside.

**Discussion:**

This trial is the first to evaluate clinical effectiveness, cost-effectiveness and implementation of screening and patient-tailored care for cognitive and emotional problems compared to care as usual in patients discharged home after ischemic stroke. Potentially, this will improve the outcomes for patients with frequently occurring cognitive and emotional problems after stroke.

**Trial registration:**

Netherlands Trial Register: NL7295, registered 25 September 2018

## Background

### Introduction

Globally, stroke ranks third in leading causes of disability-adjusted life years [1]. Following stroke, many patients experience persistent deficits and reduced functional independence: over 30% of stroke survivors have persistent restrictions in participation after 4 years [2]. As a result, stroke causes a high social and economic burden [3].

After a major stroke, patients usually receive further rehabilitation treatment [2]. On the contrary, patients with a minor stroke who have a good outcome in terms of motor functioning and communication are mostly discharged home. A substantial number of these so called ‘walking and talking’ patients do not receive treatment at outpatient rehabilitation services [4]. However, many patients with minor stroke do experience cognitive and emotional problems [4–12].

Conventional clinical measures, such as a neurological examination, the National Institutes of Health Stroke Scale (NIHSS) or the Barthel Index (BI) (an instrument to measure a person’s functioning in activities of daily living), examine cognitive and emotional functioning at a rather global level. As a result, problems in the cognitive and emotional domains may be missed by health care professionals [5–13].

Cognitive problems are, nonetheless, the strongest predictor of return to work one-year post-stroke [6]. In addition, symptoms of anxiety and depression may hinder participation in society and return to work [7–10]. These problems can be a major contributor to a decreased participation, and a diminished quality of life [4, 14–16]. While national guidelines recommend screening and follow-up care for cognitive and emotional consequences after stroke, specific advice on the timing and guidance for follow-up care is lacking [2, 17, 18]. Therefore, the scientific board of neurologists in the Netherlands appointed this topic as a top priority for further research [19].

This trial aims to examine the clinical effectiveness, cost-effectiveness and implementation of an intervention focused on screening and patient-tailored care for cognitive and emotional problems as compared to usual care in patients discharged home after ischemic stroke.

### Study objectives

The study has three objectives: clinical effectiveness, cost-effectiveness and the implementation of the intervention.

### Primary objective

#### Clinical effectiveness

To determine the clinical effectiveness of an intervention focused on screening and patient-tailored care for cognitive and emotional problems as compared to usual care on patient-reported participation in patients with ischemic stroke discharged home without follow-up treatment at an outpatient rehabilitation clinic.

### Secondary objectives

#### Clinical effectiveness

To examine clinical effectiveness of the intervention compared to care as usual in terms of cognitive complaints, depressive and anxiety symptoms, quality of life, global health, physical disability, self-efficacy and patient satisfaction with stroke-care.

#### Cost-effectiveness

To examine the cost-effectiveness of the intervention compared to care as usual in terms of costs, effects and utilities from a societal perspective.

#### Process evaluation

To examine the implementation of the intervention from the perspectives of patients and professionals.

## Methods / design

### Study design

The ECO-stroke trial is a multicenter, patient-blinded, cluster randomized controlled trial. The Medical research Ethics Committees United (MEC-U) in Nieuwegein has approved that the current study is not subject to the Medical Research Involving Human Subjects Act (reference number: W18.169). Therefore, approval will be obtained from the local medical ethics committees of all participating hospitals. At time of submission of this manuscript, approval was obtained from the local medical ethics committees of the following hospitals: BovenIJ Ziekenhuis (reference: ECO-stroke), Noordwest Ziekenhuisgroep (reference number: L019–007), Ziekenhuis Amstelland (reference: ECO-stroke), Het Van Weel-Bethesda Ziekenhuis (reference: ECO-stroke), Tergooi (reference number: 19.24), Maasstad Ziekenhuis (L2019031), Zuyderland (reference: ECO-stroke), OLVG (reference number: WO 18.135) and Gelre ziekenhuizen (reference: ECO-stroke). The study is registered in the Netherlands Trial Register (NL7295) at 2018-09-25.

### Trial center eligibility and allocation

University and non-university hospitals in the Netherlands will be invited to participate in this study. All centers that intend to participate will be examined for their current stroke care by an online questionnaire and by interviews. Centers will be excluded from the study if one of the following criteria is met:

- the current stroke care includes a screening for cognitive and emotional problems using validated screening instruments in the majority of stroke patients, within 3 months after stroke;
- the current stroke care includes long term follow-up (6 months or longer) with repeated cognitive and emotional assessments, with or without the use of validated screening instruments, in the majority of stroke patients.

After selection, the eligible centers will be randomized by an independent statistician in blocks of two in a 1:1 ratio to the intervention group or the usual care group using the web-based system Randomizer [20].

### Participants

A patient must meet all of the following criteria to be eligible to participate in this study:

- clinical diagnosis of ischemic stroke as confirmed by a neurologist after history taking, neurological examination and CT-brain or MRI-brain;
- signs and symptoms resolve sufficiently to be discharged home directly without inpatient rehabilitation or follow-up treatment at an outpatient rehabilitation clinic.

Potential patients meeting any of the following criteria will be excluded:

- age below 18 years;
- any serious comorbidity that 1) presumably interferes with the study outcomes (for example a psychiatric disorder for which supervision of a psychiatrist is needed), 2) has a progressive course (for example cancer, mild cognitive impairment or dementia) or 3) is associated with a patient’s life expectancy of less than 6 months;
- transient ischemic attack (TIA) defined as signs and symptoms that last less than 24 h and are not accompanied with ischemic lesions in the corresponding vascular territory on CT- or MRI-scan;
- hemorrhagic stroke;
- unable to understand questionnaires based on clinical judgement, for example due to insufficient command of Dutch or aphasia;
- legally incompetent on the basis of clinical judgment;
- no informed consent.

### Recruitment and consent

Within 4 weeks after the ischemic stroke, the patient will be asked to participate by his/her treating health care professional either during admission, at the outpatient neurology clinics, at the emergency room or at home by phone. All patients are free to choose whether or not to participate. If the patient agrees to take part in the study, the informed consent form will be signed.

### Blinding

Before and during the study, patients will not be informed about the treatment allocation of their hospital. Therefore, patients will be blinded during the study period. After the study patients will be informed whether or not the intervention was received. Due to the nature of the intervention and due to the cluster randomization, the researchers and the treating health care professionals will not be blinded. However, all measurements, except for the secondary outcome measurement modified Rankin Scale (mRS), will be completed by the patients without assistance of the researchers or treating health care professionals. As such, outcome measurements will be administered in a blinded manner.

### Sample size

Since no USER-Participation-R data are available concerning the participation levels of our target group, we based our sample size calculation on Cohen’s effect size as benchmark for assessing the relative magnitude of differences in participation level between the treatment arms. Although a Cohen’s effect size of 0.35 can be defined as between small and medium, such a difference in mean participation scores may be clinically important. Therefore, it was considered necessary to detect this difference in participation level between both treatment arms. A sample size of six clusters per treatment group (12 clusters in total) with 36 individuals per cluster (216 patients per treatment group; 432 patients in total) achieves 81% power to detect an effect size of 0.35 when the intracluster correlation is 0.01 using a two-sided t-test based on the number of clusters, with a significance level of 0.05 [21]. Anticipating on a 15% attrition rate in each center, we will include (36 / 0.85 =) 43 patients per center, resulting in 258 patients per treatment group and 516 patients in total.

### Intervention group

In the intervention group, patients receive an intervention that consists of a face-to-face consultation of approximately 60 min at the outpatient clinics of the neurology department in addition to usual care. This intervention will take place at approximately 6 weeks after the stroke with a maximum range of 2 weeks earlier or later. A maximum of one follow-up session after the initial consultation may be proposed by the nurse if needed, but this is not obligatory. When more follow-up sessions seem to be needed, referral for specialized (rehabilitation) care will be advised; which specialized care that will be, depends on the problems experienced and the local health care situation. The consultation will be executed by a specialized nurse, nurse practitioner or physician assistant who is experienced in and currently performs outpatient care for patients with stroke at the participating center; hereinafter referred to as ‘nurse’. If a nurse is not available in an eligible center, one of the researchers (JS) will perform the intervention.

The intervention includes:

1. a structured screening for cognitive and emotional problems using sensitive instruments: the Checklist for Cognitive and Emotional Consequences following stroke (CLCE-24), the Montreal Cognitive Assessment (MoCA) (if the MoCA has been performed at an earlier moment after stroke and the score was ≥26, the MoCA does not need to be repeated) and the Hospital Anxiety and Depression Scale (HADS);
2. a structured screening for restrictions in participation by the USER-Participation-R;
3. self-management support consisting of the following elements: a) providing timely and individualized oral and written information about stroke and its possible cognitive and emotional sequelae, b) measuring self-efficacy by the Dutch General Self-Efficacy Scale (GSES) and providing patient-tailored guidance, c) achieving shared-decision making and d) providing contact details for possible additional questions or problems;
4. a decision tool for referral to rehabilitation services based on the results of the screening instruments. Generally, if patients experience one or more participation restriction(s), as measured by the USER-Participation-R, due to the recent ischemic stroke, referral to rehabilitation services will be indicated, but is not obliged. Besides, a close cooperation with the physiatrist will be advised, in order to decide upon optimal follow-up care.

All nurses will attend a single, four-hour training to be able to perform the intervention; this training will be given by a researcher (JS). Besides, a manual will be handed out describing all components in detail. Due to the blinded nature of the trial, the manual will be published after study completion. After this training, the first intervention will be observed by the researcher. During the study, a researcher (JS) is available for consultation and further questioning on a weekly basis.

### Control group

In the control group, patients will receive care as usual according to the current protocol of the participating hospital. The local protocol probably varies among the various hospitals.

### Outcomes

Since the study has three objectives, the paragraphs ‘Outcomes’, ‘Data Collection’ and ‘Statistical Analysis’ are sorted accordingly.

### Primary outcome measure

#### Clinical effectiveness

The primary outcome measure will be the level of participation measured with the USER-Participation-R [22]. The USER-Participation-R will be completed at 6 weeks (T1), 3 months (T2) and 12 months (T3) after stroke.

### Secondary outcome measures

#### Clinical effectiveness

The following secondary outcome measures will be used: cognitive and emotional complaints (CLCE-24), depression and anxiety (HADS), quality of life (Five-Dimensional EuroQol (EQ-5D-5L)), global health (Patient Reported Outcome Measurement Information System (PROMIS) Global-10), self-efficacy (GSES) and physical disability (mRS) [23–28]. These questionnaires will be completed at T1, T2 and T3.

#### Cost-effectiveness

Quality of life will be measured with the Five-Dimensional EuroQol (EQ-5D-5L). Costs will be assessed with the Medical Consumption Questionnaire (MCQ) and the Productivity Cost Questionnaire (PCQ) [29–31]. The following cost categories will be distinguished: intervention costs, health care costs, patient and family costs and costs outside the health care sector (productivity). Data for the economic evaluation will be collected at T1, T2 and T3.

#### Process evaluation

The implementation of the intervention will be examined by a process evaluation. This process evaluation will consist of a mixed methods study, i.e. using both quantitative and qualitative methods, and will be conducted alongside the randomized controlled trial.

### Data collection

#### Demographic and stroke characteristics

In both groups, the electronic patient record will be used to complete age, sex, medical history and stroke characteristics at baseline (T0). Other demographic characteristics, such as level of education, marital status or having a partner and working status, will be registered in the questionnaires that are completed by the patients at T1.

#### Clinical effectiveness

In the centers that are randomized to the intervention group, patients will visit the neurologic outpatient clinic for the intervention approximately 6 weeks after their stroke. Patients will be asked to complete the questionnaires sent by mail and bring them along to the consultation (see Table 1). The results from the USER-Participation-R, CLCE-24, HADS and GSES will be used by the nurse for additional questioning and individualized counselling; however, the results of the completed questionnaires will not be changed. Besides, the MoCA will be completed by the nurse during the consultation (T1). The other questionnaires will be collected during the consultation, but will only be used for research purposes. Follow-up moments at T2 and T3 include questionnaires that will be sent by mail or email (see Table 1).

**Table 1:** Overview of baseline and follow-up measurements

	measurements	baseline (T0)	6 weeks (T1)	3 months (T2)	12 months (T3)
**Demographic**					
age		x			
sex		x			
education		x			
marital status / partner		x			
working status		x			
medical history		x			
**Stroke-related**					
stroke characteristics	type, hemisphere, NIHSS at admission	x			
length of hospital admission		x			
disability at discharge	mRS	x			
**Screening instruments (intervention group)**					
participation	USER-Participation-R^a^		x^a^		
cognitive and emotional complaints	CLCE-24^a^		x^a^		
cognitive disorders	MoCA^a^(not for patients in the usual care group)		x^a^		
symptoms of depression and anxiety	HADS^a^		x^a^		
self-efficacy	GSES^a^		xª		
**Primary outcome**					
participation	USER-Participation-R^a^		x^a^	x	x
**Secondary outcomes**					
cognitive and emotional complaints	CLCE-24^a^		x^a^	x	x
symptoms of depression and anxiety	HADS^a^		x^a^	x	x
physical disability	mRS	x		x	x
self-efficacy	GSES^a^		x^a^	x	x
global health	PROMIS Global-10		x	x	x
patient satisfaction with stroke-care	customized version of SASC-19			x	
quality of life	EQ-5D-5L		x	x	x
cost questionnaires	MCQ PCQ		x	x	x

*NIHSS* National Institute of Health Stroke Score; *mRS* Modified Rankin Scale; *USER-Participation-R*, Restriction subscale of the Utrecht Scale for Evaluation of Rehabilitation on the level of Participation; *CLCE-24* Checklist for Cognitive and Emotional Consequences following stroke; *MoCA* Montreal Cognitive Assessment; *HADS* Hospital Anxiety and Depression Scale; *GSES* Dutch adaptation of the General Self-Efficacy Scale; *PROMIS Global-10* Patient Reported Outcome Measurement Information System; *SASC-19* Satisfaction with Stroke-Care; *EQ-5D-5L* Five-Dimensional EuroQol; *MCQ*, Medical Consumption Questionnaire; *PCQ* Productivity Cost Questionnaire

^a^ *those questionnaires will be used as both a screening instrument and an outcome measurement*

**Fig. 1:**
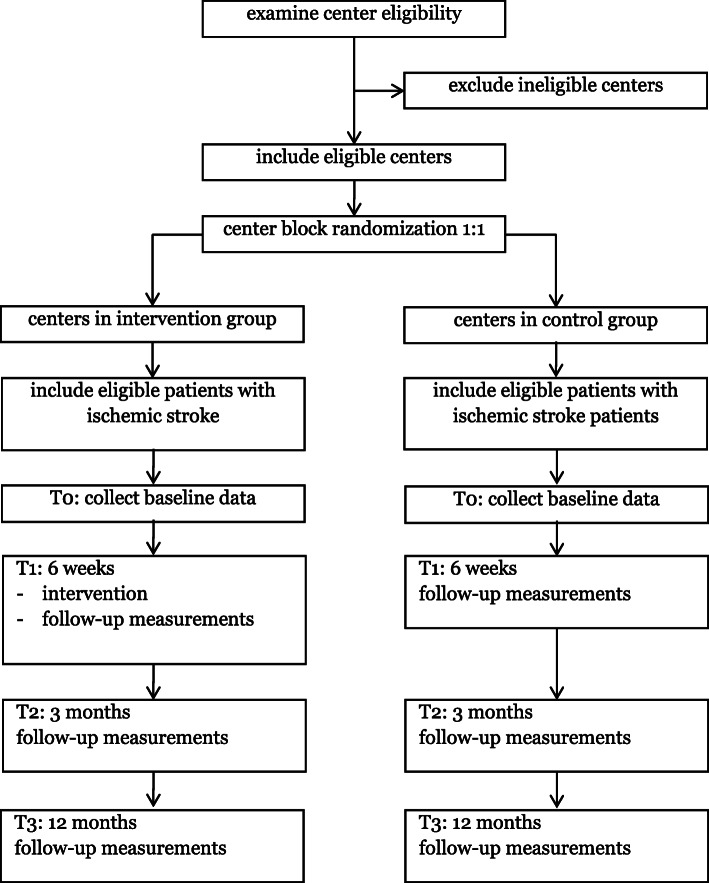
Flowchart of ECO-stroke trial procedure

In the centers that are randomized to usual care, patients will be asked to complete the questionnaires at T1, T2 and T3 (Fig. 1). These questionnaires will be sent to the patient by mail or by email (see Table 1).

The following measurements will be completed:

1. *Restriction subscale of the Utrecht Scale for Evaluation of Rehabilitation – Participation (USER-Participation-R)*

To measure restrictions in participation, the USER-Participation-R will be performed. This is a patient-reported subscale of the USER-Participation measuring experienced restrictions on 11 domains of participation, such as household activities, return to work or social activities [22]. The USER-Participation-R will be completed by the patient at home.

2. *Checklist for Cognitive and Emotional Consequences following stroke (CLCE-24)*

Subjective cognitive and emotional complaints will be measured with the CLCE-24 [23]. This checklist consists of 24 items concerning several cognitive and emotional domains. For each item the presence or absence can be scored. The CLCE-24 will be administered by the nurse in the intervention group at T1. For the control group at T1, and for all patients at T2 and T3 a self-reported CLCE-24 will be completed by the patient at home.

3. Montreal Cognitive Assessment (MoCA)

The MoCA will be administered to screen for objective cognitive impairments [29]. The MoCA assesses eight cognitive domains with a total score ranging from zero to 30. The MoCA will be administered by the nurse in the intervention group at T1, unless the MoCA has been performed at an earlier moment after stroke and the score was ≥26. The MoCA will not be administered in the control group.

4. *Hospital Anxiety and Depression Scale (HADS)*

The HADS will be administered to measure symptoms of depression and anxiety [24]. This scale consists of 14 items; symptoms of anxiety and depression will be measured by seven items each. Each item is rated on a four-point scale. The total scores of each subdomain range from zero to 21 resulting in a combined maximum score of 42. The HADS will be completed by the patient at home.

5. *Five-Dimensional EuroQol (EQ-5D-5L)*

To measure generic health related quality of life the EQ-5D-5L will be administered. The five dimensions are: mobility, self-care, usual activities, pain or discomfort and anxiety or depression. Each dimension can be rated on a five-point scale: ‘no problems’, ‘slight problems’, ‘moderate problems’, ‘severe problems’ and ‘extreme problems’ [25]. The EQ-5D-5L will be completed by the patient at home.

6. *Patient Reported Outcome Measurement Information System (PROMIS) Global-10*

To measure medical global health the PROMIS Global-10 will be administered [26]. This ten-item-questionnaire assesses global health in terms of physical health, mental health, social health, pain, fatigue and overall perceived quality of life. These ten items are to be scored on a five-point scale. The PROMIS Global-10 will be completed by the patient at home.

7. *Dutch adaptation of the General Self-Efficacy Scale (GSES)*

To measure the level of general self-efficacy the GSES will be administered [27]. The GSES consists of ten items scored on a four-point scale, ranging from ‘totally wrong’ to ‘totally true’. The GSES will be completed by the patient at home.

8. *Modified Rankin Scale (mRS)*

The mRS will be used to measure the level of disability in daily activities. The score ranges from zero (‘no symptoms at all’) to five (‘severe disability’) and six (‘dead’) [28]. The mRS will be completed by telephone with the researcher or by the nurse.

9. *National Institute of Health Stroke Scale (NIHSS)*

Stroke severity in terms of physical disability will be measured with the NIHSS [30]. The NIHSS consists of 15 items and ranges from 0 to 42. The NIHSS will be completed by the treating physician at admission in the hospital at stroke onset.

### Cost-effectiveness

All patients will be asked to complete the following questionnaires at T1, T2 and T3 together with the measurements for clinical effectiveness (see Fig. 1). These questionnaires will be sent to the patient by mail or by email (see Table 1).

The following measurements will be completed:

1. *Five-Dimensional EuroQol (EQ-5D-5L)*

The EQ-5D-5L is explained above.

2. *Medical Consumption Questionnaire (MCQ)*

Health care costs will be measured by a slightly adapted version of the MCQ [31]. The MCQ consists of 30 questions focusing on the last period, i.e. 6 weeks, 6 weeks and 9 months. The number of visits to health care professionals are registered. The type, duration and hours per day of home care is examined as well. Besides, the number of ambulance transferrals, hospital admissions and any other admission are recorded. The MCQ will be completed by the patient at home.

3. *Productivity Cost Questionnaire (PCQ)*

Family costs and costs outside the health care sector will be recorded by the PCQ [32]. The questionnaire encompasses 18 questions of which six are about demographic characteristics. The remaining questions consist of three modules, namely loss of productivity in paid work due to 1) absenteeism (absence of work) or 2) presenteeism (health related diminished-productivity at work) and 3) productivity loss in unpaid work. The questions specifically focus on productivity in the last 4 weeks. The PCQ will be completed by the patient at home.

### Process evaluation

A process evaluation was developed in accordance with the framework of the Medical Research Council and consists of the following parts: [33].

1. *Reach and Dose of the Intervention*

The reach of the intervention is defined as the number of patients actually included in the study, divided by the total number of patients that were screened for eligibility. This information will be registered in a screening log.

The dose of the intervention is defined as the number of patients actually receiving the intervention, divided by the total number of patients included in the study. This information will be registered in the electronic case report form (eCRF).

2. *Fidelity to the Intervention Protocol*

The fidelity is defined as the adherence to the original intervention protocol. The fidelity to the following components of the intervention will be registered. First, the protocol prescribes that the intervention should be performed within four to 8 weeks after the ischemic stroke. The timing of the intervention will be registered in the eCRF. Second, patient charts and eCRF will be used to register if the patient brought the completed questionnaires to their appointment. Third, in the patient charts will be registered if the nurse completed the screenings instruments during the consultation. Fourth, in the eCRF will be registered if the nurse provided individualized information and if shared decision making took place. Fifth, the nurse will register in the eCRF if the intervention could be completed within the estimated time. In addition, in a focus group interview, the nurses will be asked for experience with the application of the intervention in their daily care and the adherence to the intervention protocol for more profound understanding of possible deviations from the protocol.

3. *Contextual Factors*

Contextual factors that might affect implementation are measured by the Barriers and Facilitators Assessment Instrument (BFAI) [34]. The BFAI will be completed by the nurse after he/she delivered the intervention to ten study patients and before the focus group interview takes place. During this interview, additional information will be gathered to get a more profound understanding of the barriers and facilitators nurses reported in the BFAI during this interview for further questioning.

4. *Patient Satisfaction*

As the intervention focusses on improving care for patients with cognitive or emotional problems after ischemic stroke, participant responsiveness will be measured in terms of patient satisfaction. Patient satisfaction will be assessed by the Satisfaction with Stroke Care (SASC-19) questionnaire in the intervention group and the control group at T2 [35]. Since the first eight items of the original SASC concern the hospital admission, these items will not be administered. Two items will be added to the questionnaire, concerning shared-decision making and individualized information provision. The customized SASC-19 will be completed by the patient at home.

### Statistical analysis

#### Clinical effectiveness

Descriptive statistics will be used to describe baseline data and outcomes. Normal Q-Q Plots and the Shapiro-Wilk test will be performed to test for normality. In normally distributed continuous variables, means and standard deviations will be used. Non-normally distributed variables and ordinal variables will be expressed by medians and interquartile ranges. Categorical variables will be described by counts and percentages. Missing data will be handled by multiple imputations. Two-sided 95% confidence intervals (CI) will express the statistical uncertainty; a two-sided *p*-value < 0.05 will be considered statistically significant. Multiple testing will not be corrected for. An intention-to-treat principle will be applied to the statistical analyses.

We will perform the main analysis of the primary outcome on the change (delta) USER-Participation-R scores between 6 weeks and 12 months and a sensitivity analysis on the observed USER-Participation-R scores at 12 months. We will use a linear mixed effects model with center as a random effect and treatment arm as a fixed effect for both analyses. In addition, we will analyze the three repeated measurements (6 weeks, 3 months and 12 months) of USER-Participation-R scores using a single linear mixed effects model with nested random effects for patients within centers and treatment arm as a fixed effect.

For the continuous secondary outcome parameters (CLCE-24, HADS, PROMIS-10 and GSES) we will use the same statistical modelling approach as described above.

The continuous secondary outcome parameter SASC-19 will be analyzed using an unpaired t-test.

With regard to the ordinal scores on the mRS we will calculate the within-group median change scores as the 50th percentile of all individual differences (change from baseline to 1-year outcome assessment). Point estimate and 95% CI of the median difference in change scores between treatment groups will be calculated using the Hedges-Lehmann method [36]. Between-group difference in change scores will be analyzed using the Mann-Whitney test.

#### Cost-effectiveness

A trial-based economic evaluation will be conducted according to the Dutch guidelines and will involve a combination of a cost-effectiveness analysis (CEA) and a cost-utility analysis (CUA) [37]. In the CEA effects will be presented as clinical outcomes (i.e. USER-Participation-R). In the CUA, costs are calculated in a similar way as in the CEA, but effects are expressed in quality-adjusted life years (QALYs). Utilities will be derived from the EQ-5D-5L. To estimate the incremental cost-effectiveness, the incremental cost-effectiveness ratio (ICER) will be calculated for both the CEA and CUA. Sensitivity and uncertainty analyses will be performed. We will perform a Budget Impact Analysis (BIA) additionally to the economic evaluation according to the Mauskopf guidelines [38]. The purpose of this BIA is to estimate the financial consequences of adoption and diffusion of the intervention on a national scale.

#### Process evaluation

Quantitative data (reach and dose of the intervention, timing of the intervention, completion of the questionnaires, information provision, duration of the intervention, BFAI and SASC-19) will be analyzed using descriptive statistics. Qualitative data from the focus group interview will be transcribed verbatim. The free text will be coded and categorized for thematic analysis and will be used to analyze the experiences of the nurses from the intervention [39]. Codes will be identified, indexed and transcending themes will be identified using MAX Qualitative Data Analysis (QDA) version 2007.

## Discussion

The current protocol describes a multicenter, patient-blinded, cluster randomized controlled trial. This trial will analyze the clinical effectiveness, cost-effectiveness and implementation of structured screening and patient-tailored care for cognitive and emotional problems compared to care as usual in patients discharged home after ischemic stroke.

While previous studies have examined the effect of screening for cognitive and emotional problems in randomized trials, the majority of these studies did not examine the effect of screening for cognitive or emotional problems on its own, but as part of a larger intervention. Besides, most studies did not use validated screening instruments [36, 40–43]. To the best of our knowledge, only one earlier randomized trial examined the effect of a cognitive assessment, but did so with a rather extensive neuropsychological examination and did not plan follow-up care [44]. Nonetheless, the topics of cognitive outcome and the social aspects of living with stroke have been recognized by patients and health-care professionals as research priorities [45]. Moreover, since cognitive and emotional sequelae are highly prevalent after stroke, national guidelines already recommend an active screening for both sequelae [2, 17, 18]. Therefore, this study aims to fill an important knowledge gap.

The strengths of the study are the following. First, this study will examine the intervention in a large sample of patients in a randomized manner, which will minimize the chances of bias. Second, the local health care professionals will execute the study intervention, therewith reflecting actual clinical practice and increasing external validity as well as facilitating implementation after the trial. Although this might result in variation in conducting the intervention, thereby introducing bias, we consider this as strength since this reflects the future practice, resulting in stronger generalizability of the findings. Third, the intervention was developed by the collaboration of a multidisciplinary team and aims to further improve the collaboration between health care professionals at the neurology department and the rehabilitation department. Fourth, this study examines an important topic as prioritized by previous patient evaluations and evaluates its effectiveness based on Patient Reported Outcome Measures (PROMs) [45, 46]. Fifth, the intervention will be analyzed from both a clinical and economic perspective, and the implementation of the intervention will also be examined.

The following limitations apply to this study. First, since the intervention is a consultation, blinding of the health care professionals is not possible. However, the patient will not be informed whether or not the intervention was supplied; since most outcomes are patient-reported measurements, we expect to minimize the influence of health care professionals being aware of the allocation. Second, a cluster randomization is methodologically weaker than a patient-level randomization. However, a patient-level randomization was considered unfeasible since most hospitals have only one health care professional occupied with stroke care at the outpatient clinics. Besides, if a hospital would have two or more health care professionals providing the stroke care, contamination from the intervention-trained nurse to the untrained nurse would be likely.

In conclusion, this trial will investigate the clinical effectiveness, cost-effectiveness and implementation of active screening and patient-tailored care for cognitive and emotional problems compared to care as usual in patients discharged home after ischemic stroke.

## Data Availability

individual participant data that underlie the results reported in published articles will be shared, after de-identification, with investigators whose proposed use of the data has been approved by a review committee and who signed the data access agreement. Proposals may be submitted beginning 9 months and ending 36 months after the first article publication.

## Abbreviations

BFAI: Barriers and facilitators assessment instrument
BI: Barthel index
BIA: Budget impact analysis
CEA: Cost-effectiveness analysis
CI: Confidence interval
CLCE-24: Checklist for cognitive and emotional consequences following stroke
CT: Computed tomography
CUA: Cost-utility analysis
eCRF: Electronic case report form
EQ-5D-5L: Five-dimensional euroqol
GSES: General Self-efficacy scale
HADS: Hospital anxiety and depression scale
ICER: Incremental cost-effectiveness ratio
MCQ: Medical consumption questionnaire
MEC-U: Medical research ethics committees united
MoCA: Montreal cognitive assessment
MRI: Magnetic resonance imaging
mRS: Modified rankin scale
NIHSS: National institute of health stroke score
NTR: Netherlands trial register
PCQ: Productivity cost questionnaire
PROM: Patient reported outcome measures
PROMIS: Patient reported outcome measurement information system
QALY: Quality-adjusted life years
QDA: Qualitative data analysis
QoL: Quality of life
SASC-19: Satisfaction with stroke-care
TIA: Transient ischemic attack
USER-Participation-R: Restriction subscale of the Utrecht Scale for evaluation of rehabilitation on the level of participation
